# Data-Driven Multiresolution Camera Using the Foveal Adaptive Pyramid

**DOI:** 10.3390/s16122003

**Published:** 2016-11-26

**Authors:** Martin González, Antonio Sánchez-Pedraza, Rebeca Marfil, Juan A. Rodríguez, Antonio Bandera

**Affiliations:** Dpto. Tecnología Electrónica, University of Málaga, Campus de Teatinos, 29071 Málaga, Spain; martin@uma.es (M.G.); aspedraza@gmail.com (A.S.-P.); rebeca@uma.es (R.M.); jarodriguez@uma.es (J.A.R.)

**Keywords:** foveal images, irregular pyramids, hierarchical segmentation, visual attention, hardware/software co-design, AP SoC

## Abstract

There exist image processing applications, such as tracking or pattern recognition, that are not necessarily precise enough to maintain the same resolution across the whole image sensor. In fact, they must only keep it as high as possible in a relatively small region, but covering a wide field of view. This is the aim of foveal vision systems. Briefly, they propose to sense a large field of view at a spatially-variant resolution: one relatively small region, the fovea, is mapped at a high resolution, while the rest of the image is captured at a lower resolution. In these systems, this fovea must be moved, from one region of interest to another one, to scan a visual scene. It is interesting that the part of the scene that is covered by the fovea should not be merely spatial, but closely related to perceptual objects. Segmentation and attention are then intimately tied together: while the segmentation process is responsible for extracting perceptively-coherent entities from the scene (proto-objects), attention can guide segmentation. From this loop, the concept of foveal attention arises. This work proposes a hardware system for mapping a uniformly-sampled sensor to a space-variant one. Furthermore, this mapping is tied with a software-based, foveal attention mechanism that takes as input the stream of generated foveal images. The whole hardware/software architecture has been designed to be embedded within an all programmable system on chip (AP SoC). Our results show the flexibility of the data port for exchanging information between the mapping and attention parts of the architecture and the good performance rates of the mapping procedure. Experimental evaluation also demonstrates that the segmentation method and the attention model provide results comparable to other more computationally-expensive algorithms.

## 1. Introduction

Robotic vision needs to solve a large variety of tasks that demand different parameters. In order to deal with all of them using the same sensor, this must be adjustable to a specific task. However, it is also usual that some of these tasks run simultaneously. Thus, for instance, the module responsible for the navigation skill could need a large field of view (FoV), while the one in charge of recognizing an object could simultaneously need to capture this at a high resolution. Furthermore, this image acquisition and processing must be done quickly, since the robot must be an agent able to communicate and interact with people fluently within a dynamic world. Although it is currently possible to acquire in real time a video sequence from a high resolution sensor (up to 10 megapixels) using dedicated hardware, any algorithm that requires multiple frames will need a large off-chip memory access. This requirement will typically be the bottleneck of the framework [1].

**Figure 1:**
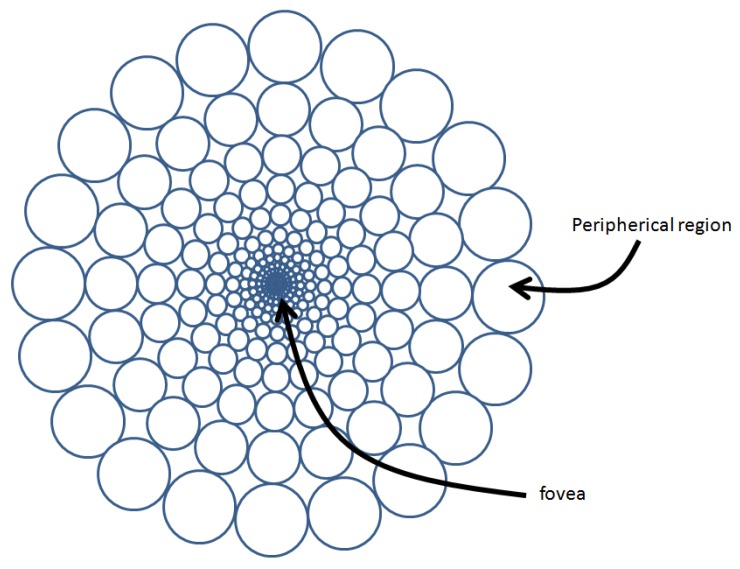
Approximated distribution of cones on the human retina, providing brain pixels of an adapted size distributed following a polar pattern (adapted from the original from C.Ware, 2008, p. 5 [2]).

The evolution of biological vision systems has reached a balance between what they can perceive and what they are capable of processing in real time. Foveal vision reduces the data volume by sampling the FoV at a variant resolution. A reduced area of the FoV, the fovea, is captured at a high resolution. This will be the area of the FoV where, for instance, we must include the objects that we want to recognize. Surrounding this fovea, the rest of the FoV is captured at a lower resolution. In fact, resolution will typically be reduced as we move away of this fovea, as Figure 1 shows. Therefore, to have a detailed vision of all of the world, we make quick movements of the eyes from one position to another.

Against this need of image detail, which provokes that the eyes make short and rapid movements while scanning a visual scene, it is also necessary to keep a record of what is happening with the rest of the FoV. As previously mentioned, this could be required by a robot navigating within a dynamic environment. In this application, the main goal is to perceive a large FoV, perhaps at lower resolution, but sufficient to be able to act if necessary.

Space-variant vision is the term used to refer to sensors that capture the image with different resolutions across the FoV, generating an image with space-variant resolution (foveal image). It reached its greatest popularity in the 1990s. Intimately tied to the concept of active perception (it does not make sense to have a fovea if the eyes cannot be swiftly moved over possible regions of interest [3]), it was the origin of several physical implementations in silicon. However, the design of a specific retina-like sensor was always a hard and expensive work. Hence, most of the proposals finally laid in a software-emulated foveal sensor, which took the input data from a uniform, real sensor [1]. Currently, this option is also supported by the fact that it is possible to acquire CMOS sensors of very high resolution at a very reduced price. On the other hand, the processing limitations of software emulation can be overcome by performing the mapping through hardware emulation. Specifically, this was a wide part of the research within our group in the past [4,5,6].

As was previously mentioned, space-variant vision is very related to the concept of active vision and also with the concept of attention, due to the necessity of moving the fovea from one region of interest to another. Attention is the process in charge of determining the region or regions of interest at each moment. Attention models are typically focused in aspects, such as the identification of features that influence the selection of the interest region, the combination of these features to generate the saliency map or how a specific task drives attention. However, they typically neglect the foveal nature of the human vision system. A significant exception is the work by Rybaka et al. [7]. This approach proposes a complex system that includes the foveation as the basis for object identification, memorizing motor and sensory inputs. The ‘what’ stream is complemented with a ‘where’ stream, which determines the location of the new focus of attention. The practical implementation of the idea of working at two different resolutions usually employs two cameras [8]: a low-resolution one for computing the saliency map of the scene by capturing a large FoV (the ‘where’ stream) and a high resolution one for studying in detail the most salient region (the ‘what’ one). This hardware layout tends to differentiate both processes. Without considering the ‘what’ stream, several authors propose foveal approaches for saliency estimation. Rajashekar et al. [9] use the foveal encoding of Geisler and Perry [10] in their gaze attentive fixation finding framework (GAFFE). It employs luminance, contrast and bandpass outputs of both luminance and contrast as low-level features to compute saliency maps and predict gaze fixations. This framework works on a sequential process in which the input image is foveated placing the fovea in the current fixation point, and the next fixation point is predicted by computing the low-level features from circular patches obtained from the foveated image. This strategy has been evaluated by Gide and Karam [11] using different sets of low-level saliency features extracted from other attention models as the attentive information maximization (AIM) or saliency using natural image statistics (SUN) [12]. To perform this evaluation, different types of distortions (Gaussian blur, white noise and JPEG compression) have been applied to input images. This work shows that foveation improves the performance of all saliency models over different types and levels of distortion.

Other authors propose to encode the foveal nature within a hierarchical structure. Advani et al. [13] organize the scene content within a three-level Gaussian pyramid in which the whole FoV is represented in the top level (with the lowest resolution), and the bottom one encodes the 50% of the field of view at the same resolution as the original image. The AIM model [14] is run at these three levels, obtaining an information map for each of them. In these maps, salient regions are represented at different resolution levels. They are combined into a unique saliency map by means of a weighted summation. Following an object-based model of attention, Marfil et al. [15] propose to estimate a saliency map using the regions generated within the foveal version of the bounded irregular pyramid (BIP), an irregular pyramid where each level is represented as a graph. Saliency estimation and segmentation are intimately tied within this work. The input data are encoded within a foveal polygon [15], a structure where the image is represented as a stack of levels of uniform resolution. The fovea is the base of the structure, and the upper levels are built by decimating the content of the level below and adding a new ring of uniform resolution. At the waist level, the structure encodes the whole field of view. Other levels can be then built over the waist, resembling the pyramid structure [15]. The main problem of this approach is that, in the foveal polygon, the whole field of view is only present at the waist level. Additionally, at this level, the resolution of the foveated portion of the scene is as reduced as the one of the peripheral region. The details are then lost.

This paper revisits the research field on space-variant, active vision, proposing a whole framework that:

- Maps the sensor data into a foveal lattice. Similar to the proposal from Martinez and Altamirano [16], this work proposes to emulate an artificial retina from a sensor of uniform resolution by a Cartesian space-variant sampling, obtaining a unique space-variant resolution image or foveal image, where the fovea has the highest resolution, and it decreases as we move away from the fovea.
- Processes this foveal image for providing a multiscale segmentation of it in order to obtain the different proto-objects present in the scene. A proto-object can be defined as regions of the image that can be bounded into an object [15].
- Performs a bottom-up attention process for choosing a new fovea.

The proposed work tests the option of embedding the complete hardware and software solution on an all programmable system on chip (AP SoC) platform. Thus, the foveal mapping is synthesized on the FPGA (programmable logic), and the multiscale segmenter and attention model are programmed on an ARM (processing system). Data between memory and modules are exchanged through direct memory access (DMA), allowing the whole framework to run at 10 frames per second (fps) (images covering a FoV of five megapixels).

### Contributions and Organization of the Paper

The main contribution of this paper is the proposal of a complete framework able to close the loop between perception and foveation. The framework is able to process 10 fps, and using foveae whose sizes are typically lesser than the 20% of the full FoV, it is able to segment natural images with a performance similar to those approaches that deal with the full FoV at uniform resolution. There are some significant theoretical aspects that must be emphasized:

1. Within an AP SoC, the hardware-emulated foveal sensor is able to provide a stream of foveal images to the software components in real time, without significant latencies. This foveal sensor is configurable at execution rates, being able to move and adapt the fovea to capture the objects of interest.
2. Contrary to previous approaches, this work suggests that the hierarchical segmentation of a captured scene can be achieved by decimating a non-uniform layout. That is, each level of our hierarchy is now a graph whose spatial sampling varies across the FoV. The typical paradigm is that a hierarchical segmentation of an input image provides a stack of successively-reduced graphs (or images) of uniform resolution.
3. The image encoding within the hierarchy of an irregular pyramid is typically performed by putting the effort on choosing the set of vertexes that, coming from a given level, will compose the level above. The vertexes at this new level will be subsequently linked among them by considering a connectivity criterion. In this work, we demonstrate that it is also interesting to consider what arcs among vertexes should not be established. Using a very simple strategy, our proposed decimation process runs significantly faster than previous approaches, exhibiting a very similar performance.
4. Using the evaluation framework provided by the BSDB500 database, this work demonstrates that it is not really needed to process the full FoV with the same level of detail. In an active scenario and after a few foveations, the proposed system is able to provide segmentation results (i.e., relevant contours) that are very close to the ones provided by human subjects.

The rest of the paper describes the proposed framework: Section 2 presents an overview of the whole framework. Then, Section 3 briefly reviews and describes the foveal lattice synthesized on the FPGA and presents the design and implementation of the chain of processing on the logic part of the AP SoC. Section 4 describes how to create the communication for the hardware cores and the ones running on the ARM. Section 5 briefly describes the hierarchical segmentation of the foveal images and the bottom-up attention process performed on the software part. Section 6 and Section 7 summarize the experimental evaluation of the proposed framework and draw the main conclusions and future work, respectively.

## 2. Overview of the Framework

**Figure 2:**
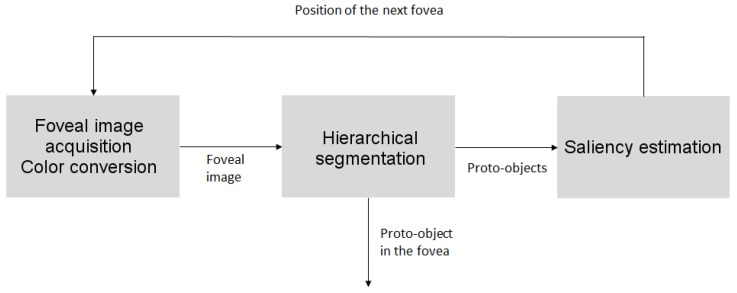
Overview of the proposed framework.

**Figure 3:**
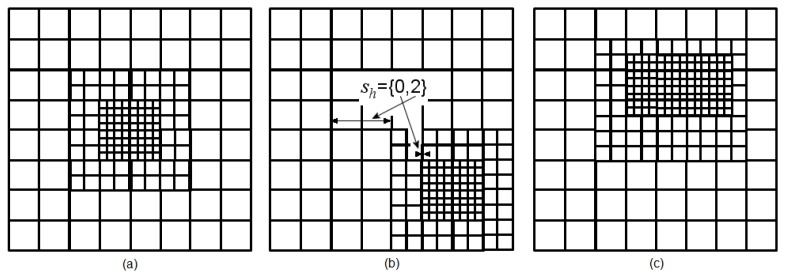
(**a**) Cartesian foveal geometry (CFG) with *m* = 3 and *d* = 2; (**b**) Shifted Fovea Multiresolution Geometry(SFMG) of generalized motion with $s_{h}$ = {0,2} and $s_{v}$ = {0,2} (we have marked in the figure the displacement among rings of resolution due to $s_{h}$: the origin of the first ring is horizontally shifted two rexels, within the second ring, and the origin of the fovea is not shifted (zero rexels) within the first ring); and (**c**) GMFD of adaptive motion with $L_{d}$ = 2, $R_{d}$ = 1, $T_{d}$ = 1 and $B_{d}$ = 3.

Figure 2 shows the main stages of the proposed framework. It must be noted that, while in an image with homogeneous resolution, the unit of information is the pixel, in a foveal image, the unit of information is the rexel or resolution cell (i.e., the brain pixels in Figure 1 or the square cells of Figure 3a–c). The first stage (foveal image acquisition and color conversion) is in charge of obtaining the uniform RGB image from the sensor, mapping this image into a foveal lattice and converting the RGB color values of the rexels to HSV color space, the one employed in our proposal. In the used foveal lattice, the fovea has the same resolution as the input image, and it decreases as we move away from the fovea following a Cartesian foveal geometry (CFG) with adaptive fovea (this will be explained in detail in Section 3.1). Then, the output of this stage is a foveal image that is represented as a graph where each vertex of the graph is a rexel of the foveal lattice. This graph is the input of the next stage of the system (the hierarchical segmenter). This hierarchy is encoded as an irregular pyramid where each level is encoded as a graph. The output of the segmenter is the set of proto-objects present in the image. Among them, the proto-object located in the current fovea, which has been captured with a high resolution, can be sent to other specific modules, i.e., object recognition. The saliency of the proto-objects is computed in the saliency estimation stage that also determines the position of the fovea in the next frame. For achieving this, the module computes the parameters that will drive the next foveation.

Apart from the theoretical contributions, the main goal of our work is the design, implementation and testing of an embedded solution for the previously-explained framework. For this purpose and given the co-existence of hardware and software modules on the whole system, we will propose to endow the architecture within an AP SoC. Specifically, we have used the Zedboard from Avnet, which is based on the Zynq-7000 AP SoC XC7Z020-CLG484-1 from Xilinx. The Zynq-7000 combines software, hardware and input/output capability over a single silicon integrated circuit. This will be the hardware base for setting our proposal. The hardware configuration for the AP SoC can be stated as the interaction between two different parts: the processing system (PS) and the programmable logic (PL). The software processing is addressed on the PS, which is mainly composed by the ARM cores, I/O peripherals, clock system generators and memory interfaces.

**Figure 4:**
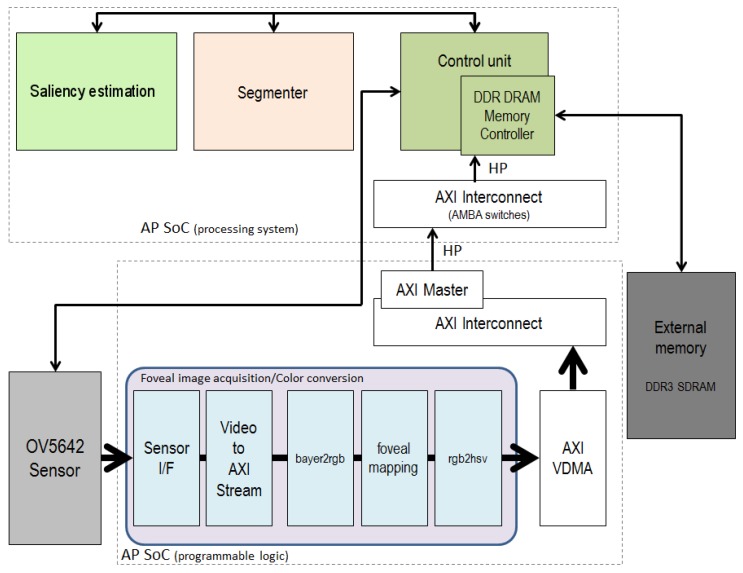
Block diagram of the system.

Figure 4 shows a block diagram of the proposed framework. In our case, the foveal image acquisition and color conversion module is synthesized in the PL, while the initial configuration of the sensor, an OV5642 of five megapixels from Omnivision, and the segmentation and saliency estimation are conducted on the PS. The figure shows that, although the initial configuration of the sensor is addressed from the PS, it accesses the sensor via the PL. The PS and PL parts are interconnected through the AXI Video Direct Memory Access (AXI VDMA) core. The AXI VDMA is a soft Xilinx IP core that provides high-bandwidth direct memory access between memory and AXI4-Stream type video target peripherals. In the figure, cores involved in the connection between the PL and the PS parts are filled with a white background. The sequence of foveal images obtained in the foveal image acquisition and color conversion module is stored in the external memory (DDR3 SDRAM) through AXI VDMA, Interconnect and high-performance (HP) interfaces/ports. From the DDR3, the segmenter takes each foveal image to be segmented. This module and the saliency estimation one have been successfully implemented on the ARM.

## 3. Foveal Mapping

### 3.1. Cartesian Foveal Geometries

Cartesian foveal geometries (CFG) encode the field of view of the sensor as a square-shaped region at the highest resolution (the fovea), surrounded by a set of concentric rings with decreasing resolution [4]. Figure 3 shows three different types of CFGs. In these geometries, each cell on the lattice is a rexel. The resolution of each rexel depends on the ring in which it is located, and it decreases from one ring to the next by a factor of four. In the majority of the Cartesian proposals, the fovea is set in the center of the geometry (fovea-centered CFG), and the rings present the same parameters (Figure 3a). Each ring is typically composed of several sub-rings. Thus, the geometry is characterized by two constant values: the number of rings surrounding the fovea (*m*) and the number of sub-rings (*d*) within each ring. The size of the fovea is stated according to *d*: if we set its dimensions to 4d×4d, there is no discontinuity between the fovea and the periphery regions.

Among other advantages, there are CFGs that are able to move the fovea through the FoV (shifted fovea). Usually, vision systems that use the fovea-centered CFG move the cameras to place the region of interest in the center of the image. A shiftable fovea is very useful to avoid these camera movements. Figure 3b shows one example of the Shifted Fovea Multiresolution Geometry (SFMG) of generalized motion [4]. Within this algorithm, each ring of resolution is shifted with respect to the center position according to two vectors ($s_{v}$ and $s_{h}$). Furthermore, the possibility to adapt the fovea to the size of the region of interest can help to reduce the consumption of computational resources [4]. Figure 3c shows an adaptive fovea. The geometry is now characterized by the subdivision factors at each side of the fovea ($L_{d}$, $R_{d}$, $T_{d}$ and $B_{d}$). This will be the geometry finally implemented within this work.

### 3.2. Hardware Implementation

Figure 4 illustrates the stages involved in the data processing within the PL. The sensor employed (OV5642) is able to provide color frames of five megapixels. The sensor I/Fcore provides the video input, consisting of parallel video data, video syncs, blanks and data validation. For bridging the video input and the video processing cores, we use the Xilinx LogiCORE^TM^ IP Video In to AXI4-Stream core. This core interfaces our video source to the AXI4-Stream Video Protocol Interface. On the one hand, this forces us to use this protocol for communicating the rest of IP cores. However, on the other hand, it simplifies the design, handling the asynchronous clock boundary crossing between the video clock domain and the processing (AXI4-Stream) clock domain.

The foveal mapping and color conversion are addressed by the three last cores on the ‘foveal image acquisition/color conversion’ block. The bayer2rgb core aims to reconstruct a color image, at the full resolution, from the commonly-used Bayer color filter array pattern provided by the sensor. Specifically, it implements a linear approach, encoded on masks of 3×3 pixels [17]. This demosaicing process provides the input data to the foveal mapping. This core is in charge of generating all rexels of the foveal lattice.

**Figure 5:**
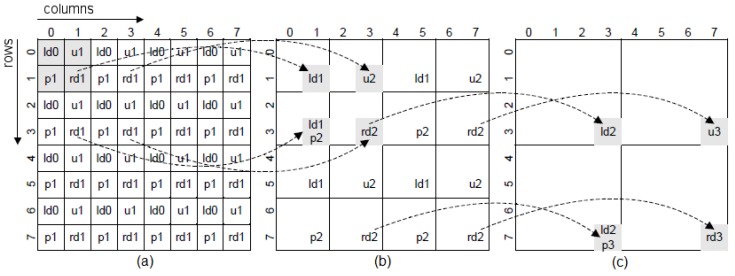
Generating the sequence of images of reduced resolution.

**Figure 6:**
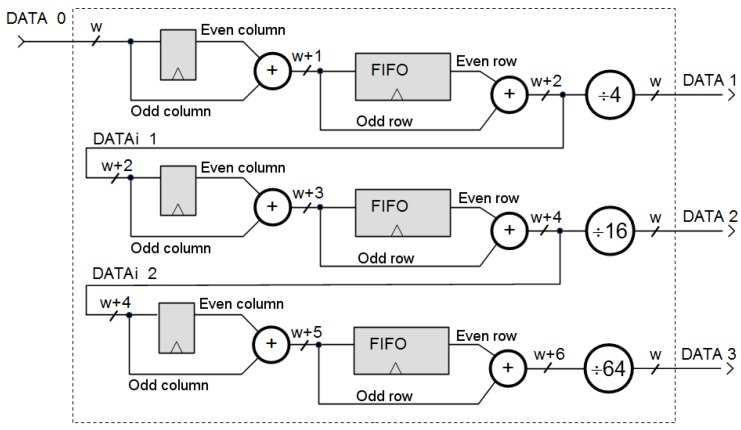
Complete structure of the datapath for generating the first three levels using data from the input image (DATA 0).

**Figure 7:**
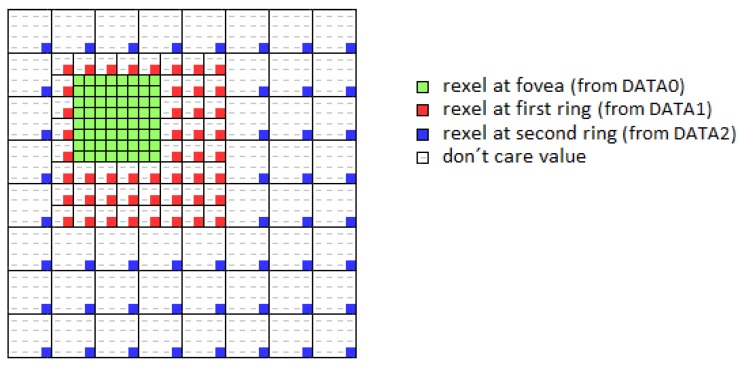
Foveal Image embedded in a full image. CFG defined by $L_{d}$ = 1, $R_{d}$ = 3, $T_{d}$ = 1, $B_{d}$ = 3. Only the fovea, the first and second rings are shown.

For achieving this foveation, we compute in parallel the rexels of all resolutions. The foveal mapping is then obtained by a sequence of four-to-one averaging stages, which will generate the complete set of rexels needed to have the foveal lattice with a CFG with adaptive fovea. It must be noted that all of these rexels are obtained at the same rate imposed by the sensor, i.e., we obtain the last rexel when the last pixel of the uniform frame is provided by the sensor. The four-to-one averaging process can be addressed in two steps. In the first one, we obtain the average of the two pixels in the upper row. In the second step, we average the two pixels in the lower row and compute the final value. The process is schematized in Figure 5. In the figure, $ldi_{i=0,1,...}$ denotes a cell that must be registered; $ui_{i=0,1,...}$ indicates the storing of the first averaging process; $pi_{i=0,1,...}$ implies the recovery of the first averaging value required for obtaining the second averaging value; and $rdi_{i=0,1,...}$ denotes the instant in which the four-to-one averaging process is complete. We have marked on the figure as gray cells these instants of interest. They are referred to the reading of the original image. Figure 5a shows the generation of the rexels with a size of 2×2 pixels. The pixel at Row 0 and Column 0 must be registered to be averaged with the next pixel in the same row. The result of this averaging process will be available at Column 1 of Row 0 and should be stored for completing the four-to-one averaging process with the two pixels at Row 1. This same process (registering, processing and storing) will be replicated for the rest of the pixels at Row 0. Thus, we will need a buffer with a length equal to the half of the width of the original image. When we will process Row 1, the values in the buffer will be extracted in the same order that they are stored. The required buffer is then a FIFO structure. The values of the rexels of a size of 2×2 pixels are obtained when we process the pixels of the input image associated with all odd columns of the odd rows (labeled with rd1 on the figure). This defines a new data flow that is processed in the same way for generating the values associated with the rexels with size 4×4 pixels. Figure 6 shows the datapath for generating the rexels of size 2×2 pixels (DATA 1), 4×4 pixels (DATA 2) and 8×8 pixels (DATA 3). The structures should be triplicated for dealing with the three channels of the RGB color encoding. It must be noted that, from the whole set of computed rexels, only some of them are part of the foveal lattice, while the rest are only needed to compute the next rexels with lower resolution. The rexel of the foveal lattice can be determined from the parameters of the CFG with adaptive fovea in the current frame ($L_{d}$, $R_{d}$, $T_{d}$ and $B_{d}$). Briefly, with this datapath structure, five streams of reduced resolution are generated. However, as we can see in Figure 7, only the rexels corresponding to different rings are needed to build the foveal image. That is, as was previously mentioned, the output of this foveal mapping is a foveal image, which is represented as a graph where each vertex is a rexel of the foveal lattice. Thus, a multiplexing stage is added after the datapath to select the rexels among the DATA i (i = 0, 1, ..., 5) streams. This multiplexing stage depends on the values of the $L_{d}$, $R_{d}$, $T_{d}$ and $B_{d}$ parameters and also of the coordinates of each pixel. The result is a unique stream, with the same size and timing as the original input image. This stream contains the foveal image required by the modules of the PS part to carry out the segmentation process. From the received image, this module only uses the colored cells in the figure. The dashed cells are discarded.

However, before sending this image to the ARM, the RGB values are translated to the HSV color space. This is achieved by the rgb2hsv core. The used conversion equations are:

(1) $$H=\left\{\begin{array}{cc} 0 & \mathrm{if}\;C_{max}-C_{min}=0,\\ 60\cdot \frac{G^{\prime }-B^{\prime }}{C_{max}-C_{min}}\;\mathrm{mod}\;\;6 & \mathrm{if}\;C_{max}=R^{\prime }\\ 60\cdot \frac{B^{\prime }-R^{\prime }}{C_{max}-C_{min}}+2 & \mathrm{if}\;C_{max}=G^{\prime }\\ 60\cdot \frac{R^{\prime }-G^{\prime }}{C_{max}-C_{min}}+4 & \mathrm{if}\;C_{max}=B^{\prime } \end{array}\right.$$

(2) $$S=\left\{\begin{array}{cc} 0 & \mathrm{if}\;C_{max}=0,\\ \frac{C_{max}-C_{min}}{C_{max}} & \mathrm{if}\;C_{max}\neq 0 \end{array}\right.$$

(3) V=Cmax

being $R^{\prime }=R/255$, $G^{\prime }=G/255$ and $B^{\prime }=B/255$, $C_{max}=\max\left(R^{\prime },G^{\prime },B^{\prime }\right)$ and $C_{min}=\min\left(R^{\prime },G^{\prime },B^{\prime }\right)$.

The obtained image should be stored in the external memory for being shared with the ARM cores. As previously mentioned, the foveal images is encoded within a uniform image whose size is the same as the one of the original image (Figure 7).

## 4. Communicating the PL and PS Cores

The proposed framework needs to handle the data stream from the PL to the DDR3 SDRAM memory at the highest possible rate. Direct memory access (DMA) will allow the rgb2hsv core to gain access to the main bus linking the processor with the DDR3 memory. This avoids the use of the ARM processor to perform load or store operations, giving the Zynq-7000 AP SoC a large I/O bandwidth and low latency in the integration of a custom logic with a custom software. Thus, once the DMA transfer has been set up by the ARM core, this will wait to be notified when a complete chunk of resolution levels is received. When a transfer is completed, the ARM core generates the foveal image and then the hierarchy of segmentation results while a new transfer is in progress. This mechanism saves CPU cycles and increases the data throughput.

**Figure 8:**
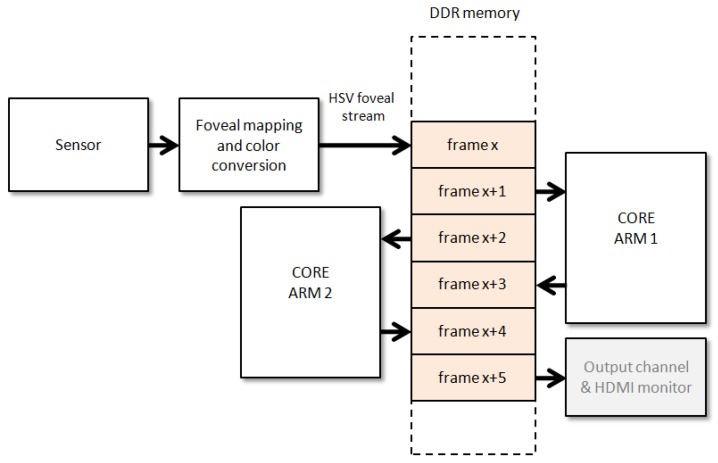
Organization of the memory space for allowing the multithread processing within the processing system (PS).

In the system block in Figure 4, the generated image encoding the foveal lattice is transferred through AXI VDMA (Video Direct Memory Access). AXI is a standardized IP interface protocol based on the ARM AMBA4 and AMBA3 AXI specifications. The AXI VDMA core can be used to transfer the AXI4-Stream protocol-based video stream to DDR memory and vice versa. AXI VDMA implements a high-performance, video-optimized DMA engine with frame buffering, scatter gather and two-dimensional (2D) DMA features. AXI VDMA transfers video data streams to and from memory and operates under dynamic software control or static configuration modes. To provide high-speed AXI master interfaces in the PL with lower latency, connections to the high performance (HP) interfaces are required. AXI Interconnect and AXI HP ports on the Zynq-7000 AP SoC together implement a high-bandwidth and high-performance memory system for use in applications where multiple devices share a common memory controller. Briefly, the AXI VDMA is connected to an HP interface by means of the AXI Interconnect and is controlled by the Cortex-A9 processor. As we can see in Figure 8, only two VDMA blocks are needed to connect the PL side with the PS side: the first one to upload the generated stream to the DDR (with the read channel disabled) and the second one to download the processed stream from the DDR (with the write channel disabled). In order to have full control of the operation of the VDMA blocks, both must operate in “park” mode. In this way we can select by software the frame-buffer we want to process. Thus, the download VDMA is always working with a buffer containing a completely processed image. The upload VDMA requires an extra SWcontrol to guarantee that no new image is received until the previous one is processed. For this purpose, the “frame counter” feature is used to determine the number of frames we want to receive. The VDMA is halted while the image processing is running and is enabled again by recharging the frame counter when the processing is finished. This strategy allows us to adjust the frame rate capture to the frame rate software processing.

Within the PS, the code is organized to run the same algorithm within two independent threads. The aim is to better exploit the power of the two cores of the Cortex-A9 processor. Furthermore, the execution of these two cores can concurrently work with the reception of a new frame. Figure 8 shows how the memory space is organized into a collection of pages. An additional subsystem can be added to visualize the segmentation/saliency results on an HDMI monitor.

## 5. Software Processing: Segmentation and Saliency Estimation

### 5.1. Hierarchical Segmentation

Segmentation is the process of partitioning an image into homogeneous regions according to some criteria. Pyramids are hierarchical structures that have been widely used in segmentation tasks [18] due to their ability to represent the image content at different resolution levels. In a pyramid, the input image is encoded using multiple representations with decreasing resolution. Therefore, segmentation algorithms based on pyramids exhibit interesting properties with respect to segmentation algorithms based on a single representation of the input image [18]: (i) the hierarchical structure of the pyramid can be adapted to the image topology using local operations; thus, global image features of interest can be detected and represented at different resolution levels; (ii) the noise in the segmentation is reduced; (iii) local and global features are computed within the same framework; and (iv) the complexity of the segmentation task is reduced due to the hierarchical nature of the pyramid.

The first attempts for developing pyramid-based approaches for image segmentation were based on setting in advance the size of all levels of the hierarchy. These regular pyramids were very efficient for solving the segmentation problem, but they were not able to correctly encode the image layout within such a rigid structure [18,19]. This rigidity problem is solved by irregular pyramids by using variable data structures and decimation processes, which allows the adaptation of the structure to the image layout.

Irregular pyramids are represented as a set of successively-reduced graphs. Each level *l* is a graph $G_{l}=\left(V_{l},E_{l}\right)$ where $V_{l}$ are the vertexes and $E_{l}$ the edges. When the input image is an image with uniform resolution, $V_{0}$ is formed by the pixels of the input image, and $E_{0}$ is defined by the four or eight neighborhood of each pixel. In the proposed work, where we are working with a foveal image, $V_{0}$ are the rexels of the foveal lattice, and $E_{0}$ represents their neighborhood relationships. In an irregular pyramid, each graph $G_{l+1}$ is built from $G_{l}$ using a decimation process. This procedure has three main stages: first, the vertexes of $V_{l+1}$ are generated by selecting a subset of surviving vertexes from $V_{l}$. Second, the non-surviving vertexes of $V_{l}$ are linked to surviving ones, generating the inter-level edges of the structure, which define the son-parent relationships. Third, the edges of $E_{l+1}$ (intra-level edges) are computed, defining the neighborhood relationships among the vertexes in $V_{l+1}$. The inter-level edges, which link a vertex $v\in V_{l+1}$ with vertexes of $V_{l}$, define the reduction window of *v*, which includes itself and all non-surviving vertexes linked to it.

The efficiency of an irregular pyramid depends on the employed decimation algorithm and also on the graph encoding used within the pyramid [18]. Among other relatively complex schemes, the data-driven decimation process (D3P), proposed by J.M. Jolion [19], uses a simple graph to encode each level of the structure and a two-step algorithm to select the set of surviving vertexes. For addressing this decimation process, the algorithm characterizes each vertex on the pyramid by the variance value $var_{i}$, estimated from the set formed by itself and its neighbors. Then, the algorithm uses two additional binary-state variables for describing each vertex *i* of a level *l*: $p_{i}^{\left(l\right)}$ and $q_{i}^{\left(l\right)}$. In the first step, the vertexes of level *l*, which are local minima according to $var_{i}$, are labeled with $p_{i}^{\left(l+1\right)}$ equal to one and $q_{i}^{\left(l+1\right)}$ equal to zero. In the second step, the algorithm only evaluates the vertexes with $q_{i}^{\left(l\right)}$ equal to one: those ones that have a local minimum ($p_{i}^{\left(l+1\right)}$ = 1) in its neighborhood are labeled with $q_{i}^{\left(l+1\right)}$ equal to zero. In the base level, the value of both variables $p_{i}^{\left(0\right)}$ and $p_{i}^{\left(0\right)}$ is set to one. For the rest of the levels, they are computed as:

(4) $$p_{i}^{\left(l+1\right)}=\left(p_{i}^{\left(l\right)}\cup q_{i}^{\left(l\right)}\right)\cap \left(var_{i}<min\left(var_{j}:\delta_{ij}^{\left(l\right)}\cdot q_{j}^{\left(l\right)}\right)\right)$$

(5) $$q_{i}^{\left(l+1\right)}=\left\{{\bar{p_{i}}}^{\left(l+1\right)}\underset{j\in N_{i}^{\left(l\right)}}{⋂}{\bar{p_{j}}}^{\left(l+1\right)}\right\}\cap \left(N_{i}^{\left(l\right)}\neq 0\right)$$

where $\delta_{ij}^{\left(l\right)}$ is equal to one if there exists an intra-level edge between vertexes *i* and *j* in level *l*, and $N_{i}^{\left(l\right)}$ are the set of neighbor vertexes of vertex *i* in level *l*.

Then, vertexes with $p_{i}^{\left(l+1\right)}$ or $q_{i}^{\left(l+1\right)}$ equal to one are the surviving ones and:

(6) $$p_{i}^{\left(l+1\right)}=q_{i}^{\left(l+1\right)}=p_{i}^{\left(l+1\right)}\cup q_{i}^{\left(l+1\right)}$$

**Figure 9:**
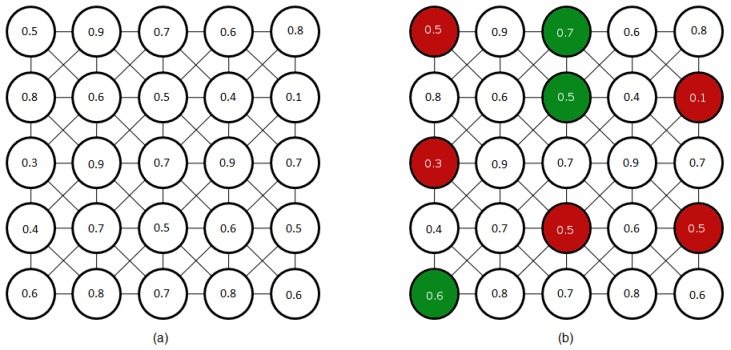
(**a**) Original graph (within each vertex, we have annotated its $v_{i}$ value); and (**b**) surviving vertexes marked in red (local minima) and green.

The surviving vertexes define $V_{l+1}$. Then, a vertex $v_{i}$ of $G_{l}$ survives if and only if it is a local minimum or does not have any surviving vertex in its neighborhood. Figure 9 provides a simple example. Red vertexes are local minima: their $var_{i}$ value are lesser than the $var_{j}$ values of their neighbors. Green vertexes do not have any local minima in their neighborhoods. They will survive without taking into consideration their $var_{i}$ values. The surviving of these last vertexes is not iteratively evaluated. This is the reason for the two adjacent green survivors in Figure 9b.

**Figure 10:**
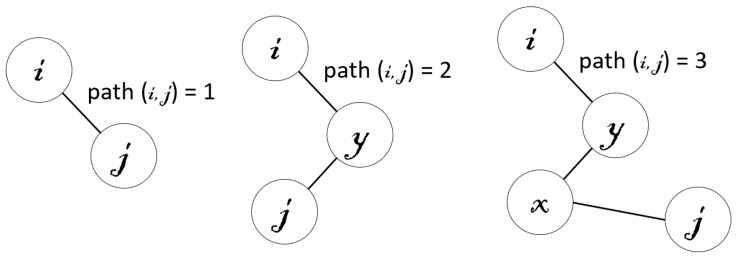
Graphical illustration of the three path concepts (1,2,3).

After choosing the survivors, the intra-level edges at this new level, $E_{l+1}$, are obtained as:

(7) $$E_{l+1}=\left\{\left(i,j\right)\in V_{l+1}\times V_{l+1}:i\neq j\cap path\left(i,j\right)\leq 3\right\}$$

where the path between to vertexes *i* and *j* is equal to one if these vertexes are linked by an intra-level edge at level *l*. This path is equal to two if there is a non-surviving vertex *y*, which is linked by intra-level edges with *i* and *j* at level *l*. Additionally, this path is equal to three if there are two non-surviving vertexes *y* and *x* that satisfy that, at level *l*, *y* is liked by intra-level edges with *i* and *x*, and *x* is linked by an intra-level edge with *j* (see Figure 10 for a graphical illustration):

(8) $$path\left(i,j\right)=1\Leftrightarrow \delta_{ij}^{\left(l\right)}$$

(9) $$path\left(i,j\right)=2\Leftrightarrow \exists y\in V_{l}:\delta_{iy}^{\left(l\right)}\cap \delta_{yj}^{\left(l\right)}\cap y\notin V_{l+1}$$

(10) $$path\left(i,j\right)=3\Leftrightarrow \exists y,x\in V_{l}:\delta_{iy}^{\left(l\right)}\cap \delta_{yx}^{\left(l\right)}\cap \delta_{xj}^{\left(l\right)}\cap y,x\notin V_{l+1}$$

Inter-level edges, linking each non-surviving vertex with a surviving one at level *l* + 1, are established by imposing that both vertexes must be neighbors at level *l*.

#### The Bounded D3P

The main advantage of the D3P is that, being very simple, it provides results comparable to other irregular approaches (see Section 6). However, there are two main problems related with its implementation. On the one hand, it does not bound the number of neighbors of the vertexes. This makes it impossible to bound the computational times and memory resources. On the other hand, the estimation of the path(i,j) values in Equations (9) and (10) is a very hard problem, as it is needed to evaluate all possible paths in level *l* for obtaining $E_{l+1}$. This computation must be iterative, provoking that the whole algorithm will be slow.

This paper proposes to address both problems, alleviating the computational cost and memory resources needed to run the algorithm. This new version, the bounded D3P (BD3P), takes into account the final task for reducing the number of edges on $E_{l+1}$ and speeding up its computation. The first issue is addressed by removing from the neighborhood of vertex *x* those vertexes whose color is very different from the color of *x*. This qualitative assertion is implemented by a simple thresholding. Thus, the neighborhood $N_{x}$ of a vertex *x* is now defined as:

(11) $$\left\{y\in N_{x}:\delta_{xy}\cap d\left(x,y\right)<t_{c}\right\}$$

where d(x,y) defines the HSV color distance between vertexes *x* and *y* and $t_{c}$ is a threshold value. Thus, Equations (4) and (5) are now defined as follows:

(12) $$p_{i}^{\left(l+1\right)}=\left(p_{i}^{\left(l\right)}\cup q_{i}^{\left(l\right)}\right)\cap \left(var_{i}<min\left(var_{j}:\left(\delta_{ij}^{\left(l\right)}\cap d\left(i,j\right)<t_{c}\right)\cdot q_{j}^{\left(l\right)}\right)\right)$$

(13) $$q_{i}^{\left(l+1\right)}=\left\{{\bar{p_{i}}}^{\left(l+1\right)}\underset{j\in N_{i}^{\left(l\right)}:\delta_{ji}\cap d\left(j,i\right)<t_{c}}{⋂}{\bar{p_{j}}}^{\left(l+1\right)}\right\}\cap \left(\left\{j\in N_{i}^{\left(l\right)}:\delta_{ji}\cap d\left(j,i\right)<t_{c}\right\}\neq 0\right)$$

**Figure 11:**
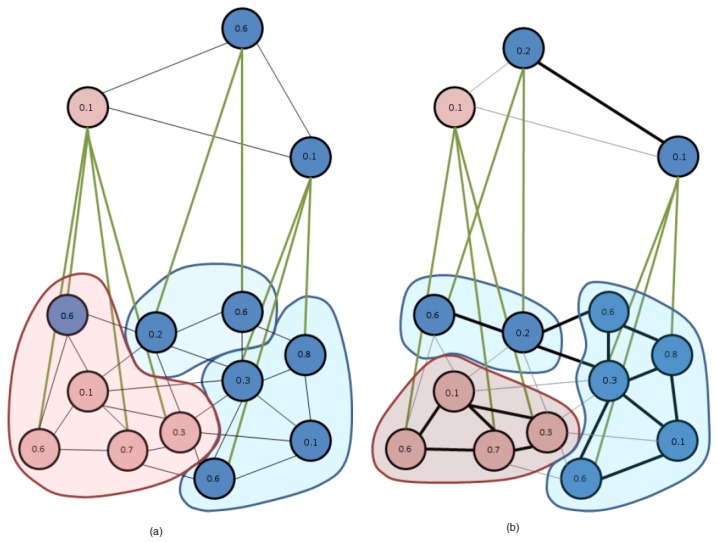
Influence of the thresholding of intra-level edges within the internal structure of the data-driven decimation process (D3P) algorithm: (**a**) The original D3P; and (**b**) The bounded D3P (BD3P) (see the text for details).

The effect of this thresholding is illustrated in Figure 11. Figure 11a provides a naive example of the application of the D3P algorithm to a set of 11 vertexes. When the rules for obtaining the surviving vertexes are applied, the algorithm chooses three survivors. The color of one of them is light gray (red), and the color of the other two is dark gray (blue). When the non-surviving vertexes look for a surviving one for establishing the inter-level edges, one of the blue vertexes (with a 0.6 value) must link to the red survivor, as this is the only surviving vertex within its neighborhood. This linking clearly disturbs the definition of the reduction window of the red surviving vertex (composed by four red vertexes and one blue vertex). Figure 11b shows the application of the BD3P to this same set of vertexes. Assuming that $t_{c}$ avoids the linking of red and blue vertexes, the neighborhood of each vertex is now quite different. We have marked as bold lines this new set of intra-level edges on level *k*. There are again three surviving vertexes at level *k* + 1, but they are different from the previous case (the 0.6-valued vertex is substituted by the 0.2-valued one). All non-surviving vertexes must now find a survivor within their neighborhood, but intra-level edges at level *k* impose that linked vertexes are of the same color. Subsequently, the three reduction windows are now composed by vertexes of the same color.

With respect to the second issue, the removal of the computation of the path(i,j) values in Equations (9) and (10) is achieved by determining that there will be an edge between two vertexes *x* and *y* at level *l* + 1 if the reduction windows of these two vertexes are in contact at level *l* (and if $d\left(x,y\right)<t_{c}$). Thus, $E_{l+1}$ can be computed by a single evaluation of the vertexes at level *l*.

### 5.2. Estimating the Salience

Once the image has been decomposed within a set of regions (our proto-objects), the next step is to choose the most relevant one. This region will be the focus of attention, conditioning the subsequent foveation and segmentation processes. Following the general rules proposed by Jeremy Wolfe and Todd Horowitz [20], we have chosen five low-level features for guiding attention. Two main guidelines have been taken into account in this selection: attention guidance and computational cost. Regarding attention guidance, Jeremy Wolfe proposes four features as undoubted to guide attention: color contrast, motion, orientation and size. From this set of attributes, we have selected the color contrast, size and orientation features. Motion has been discarded as an attribute due to its high computational cost. Another used feature is the intensity contrast value, which is a special case of the color contrast feature (it deals with the case of the gray values, including white or black). Finally, we have added a feature more related with the shape of the proto-object: the roundness, which is a measure about the closure and the contour of an object. It has been classified as a probable attribute to guide attention in [20].

Size has been selected as a direct indicator of the probability that the region belongs to the background because larger regions usually correspond with surfaces without relevance, such as walls or floors. Furthermore, it is used to discard regions of a very small size, which will normally appear in the fovea or close to it (as the segmentation of this area maintains minor details). It has been computed as a size contrast measure. That is, it is obtained from the comparison with the mean area of the regions on the image, $\bar{area}$

(14) $$SizeCON_{i}=\left|area_{i}-\bar{area}\right|$$

being $area_{i}$ the area of the proto-object $P_{i}$.

Color and intensity (brightness) contrast values measure how different a region is with respect to the ones in its surroundings according to its color and brightness. The influence in the guidance of the attention of a region is proportional to its contrast values. The more different in color and intensity is a region, the more influence it has in the guidance of attention. The color contrast feature is computed as:

(15) $$ColCON_{i}=\frac{S_{i}}{b_{i}}\sum_{j\in N_{i}}b_{ij}\cdot disColor\left(C_{i},C_{j}\right)$$

being $N_{i}$ the set of proto-objects that are neighbors of $P_{i}$, $b_{ij}$ the arc length between proto-objects $P_{i}$ and $P_{j}$ and $b_{i}=\sum_{\forall j\in N_{i}}b_{ij}$ (perimeter of $P_{i}$). $disColor(C_{i},C_{j})$ is the HSV distance between the mean color values of the proto-objects $P_{i}$ and $P_{j}$, and $S_{i}$ is the mean saturation value of $P_{i}$. To avoid the intermediate computing of the $b_{ij}$ values, the feature can be calculated at the rexel level. This allows us to compute the contrast by analyzing the set of rexels of the proto-object:

(16) $$ColCON_{i}=\frac{S_{i}}{b_{i}}\sum_{j\in M_{i}}\sum_{k\in \frac{N_{j}}{M_{i}}}b_{jk}\cdot disColor\left(C_{j},C_{k}\right)$$

$M_{i}$ defining the set of rexels that compose $P_{i}$ and $N_{j}$ the neighbors of rexel *j*. The value $b_{jk}$ is equal to the length value of the side of a rexel (a known value). The color value of the rexel, $C_{j}$, coincides with the color value of $P_{i}$.

Due to the use of the saturation value $S_{i}$ on the previous equations, the proto-objects whose color value is close to white, black or gray are not considered as relevant regions. The intensity contrast feature tries to compensate this effect. The computation is similar to Equation (16):

(17) $$IntCON_{i}=\frac{1}{b_{i}}\sum_{j\in M_{i}}\sum_{k\in \frac{N_{j}}{M_{i}}}b_{jk}\cdot disBrightness\left(I_{j},I_{k}\right)$$

but now, we employ the mean brightness values of the rexels and do not take into consideration the saturation value $S_{i}$.

The roundness feature measures the similarity of the proto-object with a circle. In our case, it is computed using the classical approach based on moments. Specifically, we use three central moments:

(18) $$\mu_{1,1}^{i}=\sum_{i}w_{i}\left(x_{i}-\bar{x}\right)\left(y_{i}-\bar{y}\right)\;\;\;\forall \left(x_{i},y_{i}\right)\in P_{i}$$

(19) $$\mu_{2,0}^{i}=\sum_{i}w_{i}{\left(x_{i}-\bar{x}\right)}^{2}\;\;\;\forall \left(x_{i},y_{i}\right)\in P_{i}$$

(20) $$\mu_{0,2}^{i}=\sum_{i}w_{i}{\left(y_{i}-\bar{y}\right)}^{2}\;\;\;\forall \left(x_{i},y_{i}\right)\in P_{i}$$

being $\left(\bar{x},\bar{y}\right)$ the center of mass of the proto-object $P_{i}$.

Using them, it is possible to evaluate the eccentricity, i.e., the difference between the region and a perfect circle:

(21) $$ecc_{i}=\frac{{\left(\mu_{2,0}^{i}-\mu_{0,2}^{i}\right)}^{2}+{\left(2\cdot \mu_{1,1}^{i}\right)}^{2}}{{\left(\mu_{2,0}^{i}+\mu_{0,2}^{i}\right)}^{2}}$$

This result is bounded to the range [0...1]. The roundness is easily obtained from the eccentricity value $ecc_{i}$:

(22) $$ROUND_{i}=1-ecc_{i}$$

The orientation of a region can be evaluated using the central moments:

(23) $$\phi_{i}=\frac{1}{2}atan\left(\frac{2\mu_{1,1}^{i}}{\mu_{2,0}^{i}-\mu_{0,2}^{i}}\right)$$

However, by itself, this measure does not reflect the relevance of the region. It will be similar to using the color of the proto-object to determine its importance. For providing a salience measure, the orientation of the proto-object must be compared with the one of the rest of the regions in the scene; that is, to estimate an orientation contrast. This measure is obtained using:

(24) $$OriCON_{i}=\sum_{j}\left|\phi_{i}-\phi_{j}\right|$$

being *j* the set of regions that compose the image.

The final saliency of a region, $sal_{i}$, is determined by the weighted sum of these five features:

(25) $$sal_{i}=\vec{\lambda }\cdot \vec{f}$$

$\vec{\lambda }$ being the set of weights, which will be normalized ($\sum_{i}\lambda_{i}=1$), and $\vec{f}$ is the feature vector. All features will be also normalized to be in the range [0...255]. Thus, saliency values will be normalized to this same range and could be visualized as gray-scale images. The set of weights to be used depends on the final application of the proposed attention system; specifically, it depends on the type of proto-objects needed by this final application. Therefore, it can be seen as a top down modulation of the attention. When this modulation is not necessary because a general scene exploration task is performed, these weights have all the same value (0.2).

#### Influence of the Foveal Lattice on the Salience Estimation

**Figure 12:**
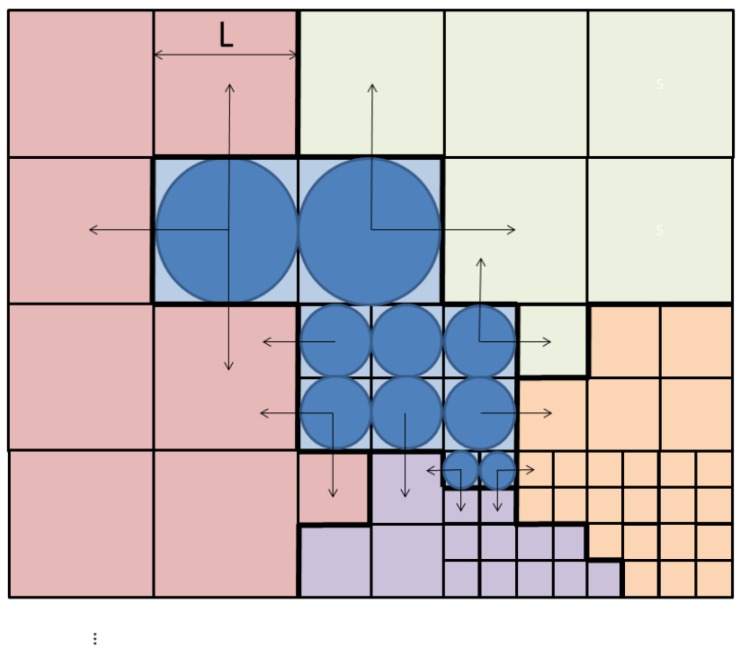
The region on a foveal image could be composed of rexels of different sizes. This will influence the computation of perimeters, means or moments (see the text for details).

In the previous section, the different feature values used in our model were presented. Their equations include perimeters, means or moments. It is important to note that our foveal lattice will impose several requirements on these properties. For describing the problem, we will use as an example the blue region in Figure 12. This region is composed by rexels of different resolution. Additionally, there is no pixel-based, uniform representation. Thus, for instance, the perimeter of this blue region, or the length of this perimeter shared with each one of the four regions on its vicinity, cannot be computed by adding pixels. The perimeter $b_{i}$ of the blue region can be computed by:

(26) $$b_{i}=5\cdot L+7\cdot L/2+4\cdot L/4$$

being dependent on the size of the rexels (in this case, *L* is the side of the largest rexel within the blue region). We should take this issue into account when we store the neighbors of each region. Furthermore, as the BD3P algorithm does not store the whole neighborhood of the node (but only those that are similar in color), we should memorize a new field for each node where storing all of this information.

When we compute the centroid $\left(\bar{x},\bar{y}\right)$ of a region, we should also take into account the different area of each one of the rexels that compose the region. That is:

(27) $$\left(\bar{x},\bar{y}\right)=\frac{1}{\sum_{i}w_{i}}\sum_{i}w_{i}\left(x_{i},y_{i}\right)$$

being $w_{i}$ the weight (i.e., the area) associated with each rexel $\left(x_{i},y_{i}\right)$. The $\left(x_{i},y_{i}\right)$ value is the center of mass of the rexel.

Finally, the computation of each moment should be also weighted by this factor. For instance, for the moment $\mu_{1,1}$:

(28) $$\mu_{1,1}=\sum_{i}w_{i}\left(x_{i}-\bar{x}\right)\left(y_{i}-\bar{y}\right)$$

## 6. Experimental Results

### 6.1. Performance and Utilization of the PL Cores

The image acquisition, foveal mapping and color conversion were synthesized on the programmable logic of the Zedboard. Table 1 shows the system resource utilization for providing these functionalities. The loop latency shows the time required by each core to process the full image (the image size is 2592 × 1944 (5,038,848 pixels)). The AXI stream interface is used for transferring the image data. This interface allows an IP core to start working before the previous core on the processing chain finishes the processing of the whole image. Thus, the latency value between the input of the first pixel of a frame to the first core and the output of this pixel to the last core is 5,227 clocks. Additionally, this value is mainly due to the line buffer employed by the demosaicing process. The pipeline structure ensures an initiation interval equal to one, i.e., the PL cores are able to process a new pixel datum with each clock cycle. Briefly, each frame provided by the sensor is processed before a new one starts to be acquired. Taking into account the latency, the preprocessing chain is almost transparent to the full architecture.

### 6.2. Evaluation of the Segmentation Algorithm

Table 2 assesses the BD3P with other decimation approaches using 50 color images from the Coil-100 database. Images have been resized to 256 × 256. Among the approaches, there are regular ones, such as the linked pyramid (LRP) [21] or the probabilistic one with a weighted relinking process (WRP) [22], and irregular approaches, such as the data-driven decimation process (D3P) [19], the bounded irregular pyramid (BIP) [18], the hierarchy of partitions (HIP) [23] and the combinatorial pyramid (CIP) [24]. The LRP [21] is built using an iterative process in which in the first iteration, each 4×4 set of vertexes of a level *l* generates a new vertex in level l+1 by averaging their image values (e.g., gray level). The set of 4×4 windows are 50% overlapped (4×4/4 pyramid), and then, each vertex in level l+1 has a set of 4×4 candidate sons at level *l* and a set of 2×2 candidate parents at level l+2. In each iteration, the whole structure is covered, and each vertex is linked to its most similar candidate parent. After that, the values of the parents are recalculated. This process ends when there are no modifications in the son-parent links between two consecutive iterations. The WRP [22] is similar to the LRP, but each vertex maintains all of the links with its candidate parents. These links are weighted edges, and the value of each weight depends on the son-parent similarity. The BIP [18] is a mixture of regular and irregular data structures: a 2×2/4 incomplete regular structure and a simple graph. The regular structure is named incomplete because, although the whole storage structure is built, only the vertexes with sons that are homogeneous in the used image value are set in it. The irregular part is built using a union-find strategy. The idea of HIP [23] is to build a minimum spanning tree (MST) of the input image to find the region borders in a bottom-up way using a dual graph structure. In the CIP [24], the pyramidal structure is represented using a hierarchy of combinatorial maps, which is built following a union-find strategy. These approaches have been compared using the *Q* function, an estimator of the segmentation accuracy [18], which takes into account the following:

- Obtained regions must be uniform and homogeneous.
- Regions must not have small holes inside.
- Neighbor regions must be significantly different according to the image value used during the segmentation process.
- Regions must not have a small size.

The smaller the value of *Q*, the better the segmentation is. All of the approaches shown in Table 2 have been tuned for obtaining the best score on the *Q* parameter:

(29) $$Q\left(I\right)=\frac{1}{1000\cdot N\cdot M\sqrt{R}\sum_{i=1}^{R}\left[\frac{e_{i}^{2}}{1+logA_{i}}+{\left(\frac{R(A_{i})}{A_{i}}\right)}^{2}\right]}$$

being M×N the size of the image and *R* the number of segmented regions. $A_{i}$ and $e_{i}$ are the area of the region *i* and its average color error, respectively. Color error in the RGB space is computed as the Euclidean distance between the color components of each pixel of the original image and the components of the color of the segmented region. $R(A_{i})$ is the number of segmented regions with area equal to $A_{i}$.

In Table 2, the minimum, maximum and average values of *Q* ($Q_{min}$, $Q_{max}$ and $Q_{ave}$, respectively) obtained from the 50 color images of the Coil-100 database are shown. Furthermore, the minimum, maximum and average structure height ($h_{min}$, $h_{max}$, $h_{ave}$) are presented. This table shows that the results provided by the BD3P are comparable to the ones obtained by the best approaches. However, it is able to run at less than 100 ms on the Cortex-A9 processor.

The BD3P algorithm has been also evaluated using the precision-recall metric and the database of natural images proposed by the group of Prof. Jitendra Malik from the University of Berkeley (BSDB500) [25,26]. Precision (P) measures the percentage of boundary pixels in the obtained segmentation that match the boundary pixels in the ground truth. Recall (R) measures the percentage of boundary pixels of the ground truth image that are in the segmented image. Both measures are independent of the input parameters used by the segmentation methods. Our algorithm has two main parameters: the $t_{c}$ value employed for thresholding the intra-level edges; and the maximum number of neighbors $n_{max}$ for the vertex. This second parameter has been set for providing a maximum bound to the number of neighbors. This section analyzes how to choose correct values for these parameters and what results are obtained by using the BSDB500 dataset (for foveal processing, the images on this dataset will be converted from a 481 × 321 size to 480 × 320). The width and length of the FoV should be a multiple of the side of the maximum rexel on the lattice (in our case, 32 × 32 pixels).

**Figure 13:**
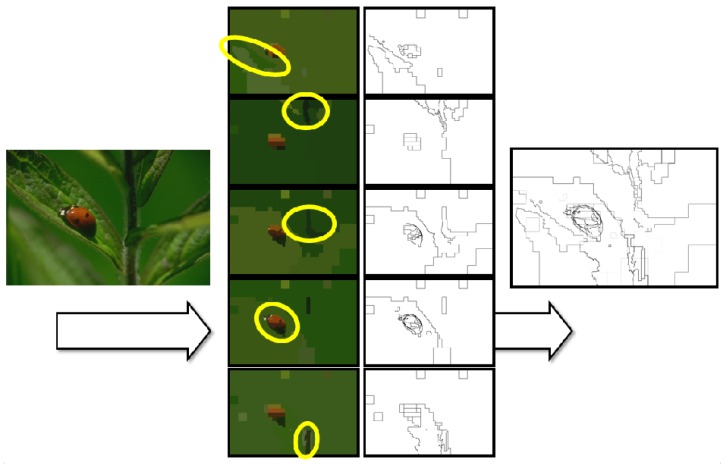
Obtaining the contour map associated with an input image as a combination of five foveations.

On the other hand, it is important to determine how the segmentation scheme applies to a static image. That is, as we mentioned in Section 1, the use of a foveal strategy does not make sense if the fovea cannot be swiftly moved over possible regions of interest [3]. For verifying the performance of the foveal segmenter, we firstly obtain a segmentation result centering the fovea in the image. The saliency of the regions composing this segmentation image is evaluated (with each weight $\lambda_{i}$ set to 0.2), and we choose the five most salient regions. Once these regions are chosen, we segment the image by setting the fovea over each one of these regions. Figure 13 schematizes the procedure. The borders within each segmentation image are obtained and labeled according to the rings where they are detected (strong borders are associated with rexels of a minor size). This provides us five sets of contours per input image. They are finally combined in the contour map that is evaluated using the precision-recall metric.

#### 6.2.1. Choosing the Parameters

**Figure 14:**
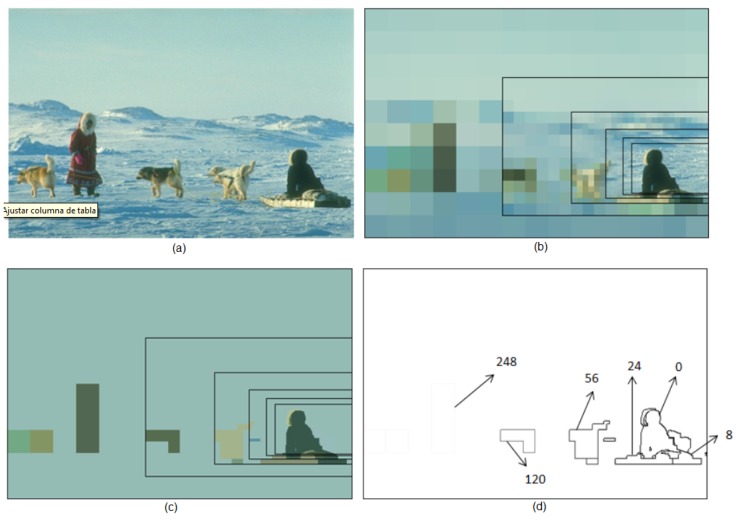
(**a**) Image #310007 of the BSDB500; (**b**) One foveal image; (**c**) Segmentation image; and (**d**) Associated contour map.

The BSDS500 provides a set of images for learning and a different set for testing. Using the first set, we have set the $t_{c}$ value to 200 and the $n_{max}$ value to 10. With respect to the sensibility of the approach to changes on the parameters, we can conclude that setting the $n_{max}$ value to 10, the $t_{c}$ value can range from 150 to 250, and the recall value typically remains over 0.9. When the $t_{c}$ value is greater than 250, the recall value diminishes as some real contours are lost. When the $t_{c}$ value is lower than 150, the number of vertexes on each level of the hierarchy grows and, with them, the height of the pyramid and the space occupied by the structure on the system memory. Furthermore, the precision value decreases, as contours that are not present in the human segmentation arise. In fact, if the recall value maintains high values relatively easily, this will not be the same for the precision value. Figure 14 shows that the approach does not only provide the real contours, strongly marked when the fovea is close to the regions where they are, but also other contours, weakly marked on these same regions, which will be generated when these regions are perceived by the peripheral vision. The precision value decreases because of these last contours as, being for instance defined by rexels of 32 × 32 pixels, they are not really very precise. To compensate for this effect, we propose to label these contours by lower values (see Figure 14).

#### 6.2.2. Evaluation on the BSDB500

In order to perform the evaluation of a segmentation algorithm using the precision and recall approach [25,26], it is needed to threshold the obtained contour map using different threshold values. For each threshold, the contour map is compared with the ground truth data (a collection of human-marked boundaries), obtaining its precision and recall values. This set of precision and recall values forms the precision and recall curve. Therefore, this curve shows the trade-off between misses and false positives (the higher the value of precision, the smaller the number of false positives, and the higher the value of recall, the smaller the number of missed positives).

**Figure 15:**
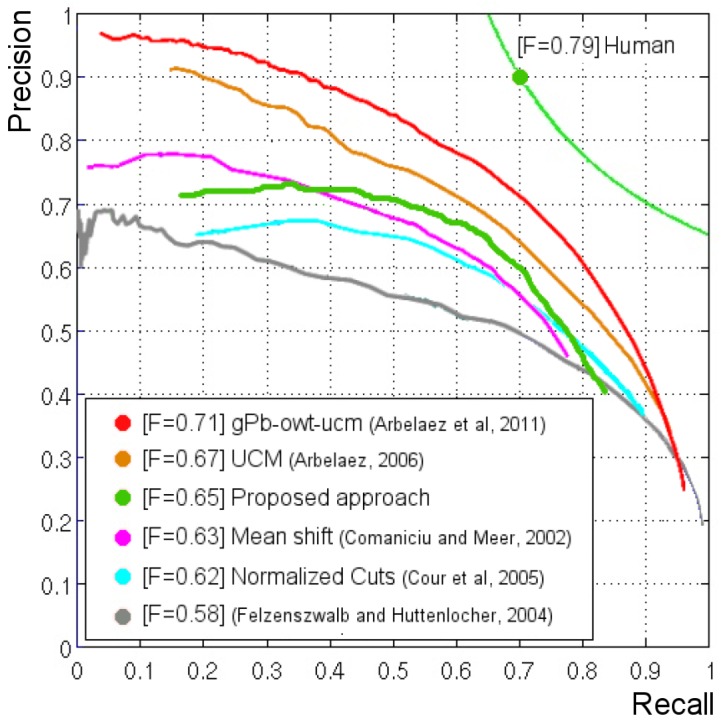
Comparison of our segmentation proposal with other approaches. The data to compute the precision-recall curves for these other approaches have been downloaded from [26].

Figure 15 compares the performance of the proposed segmentation approach with respect to the ones provided by other approaches. Precision and recall values can be combined in a unique measure (the F-measure), defined as their harmonic mean. For each algorithm, the maximum obtained F-value is shown. The light green line of Figure 15 is the function F(P,R) obtained comparing the human segmentations among them. It represents the reference with respect to which the performance of the different segmentation algorithms has been evaluated. Our approach is overcome by the gPb-owt-ucm [26] and the Ultrametric Contour Map (UCM) [27], obtaining better results than other methods [28,29,30]. However, it is important to note that our approach is processing these images to more than 10 fps. As five foveations are used (see Section 6.2), we are able to provide the contours of the image in less than 500 ms. In a real scenario, the system will not need to perform these static foveations. In less than 100 ms, the fovea will be moved through the FoV to the next salient region. It is important to note that we are able to provide similar results as those approaches that process the entire FoV at uniform resolution.

### 6.3. Evaluation of the Attention Model

**Figure 16:**
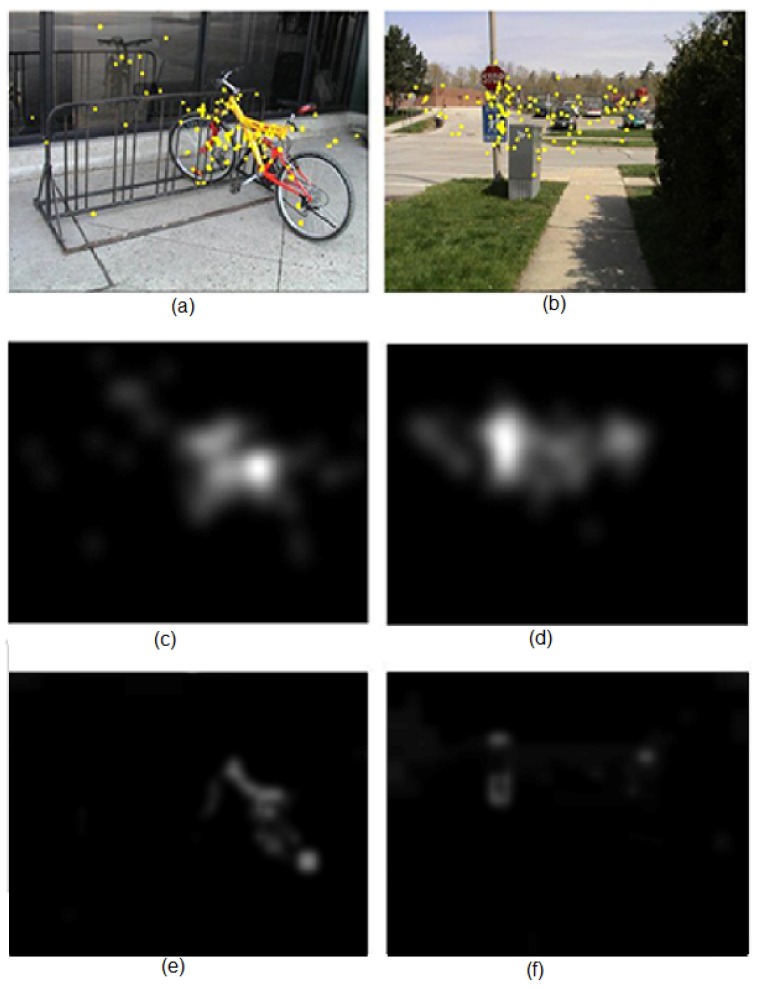
(**a**,**b**) Images from the Toronto database, annotated with the eye fixation points (see the text for details); (**c**,**d**) Density maps obtained from the fixation points from 20 people; and (**e**,**f**) Density maps obtained using the proposed framework.

**Figure 17:**
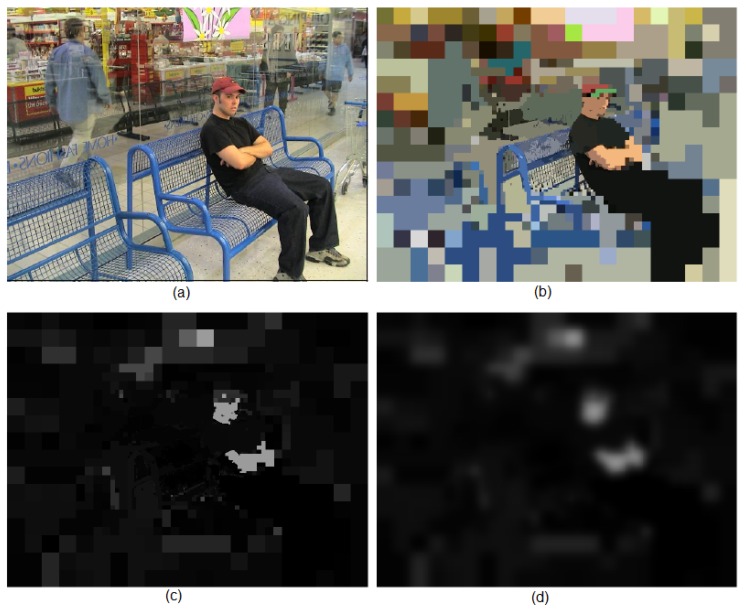
(**a**) Image #67 of the Toronto database; (**b**) Segmentation provided by the BD3P (centered fovea of 176 × 140 pixels and five resolution rings); (**c**) Saliency map provided by the attention model (λ={0.2,0.2,0.2,0.2,0.2}); and (**d**) Density map obtained by Gaussian smoothing (σ=10.0).

The attention model was evaluated using the Toronto database [14], the most popular one according to the review paper by Ali Borji and Laurent Itti [31]. This dataset consists of 120 images (681 × 511 pixels) with information of eye tracking from 20 people. The scenario is a free-viewing one, i.e., any specific task was not set, such as face or person detection. People looked at the image for four seconds. Figure 16a,b shows two images from the database. Eye fixations have been drawn over the images. Based on these fixation points, it is possible to obtain a density map for each image [14]. We illustrated two of them in Figure 16c,d. These ground truth saliency maps are basically built by inserting a value of one at eye fixation points and convolving the result with a Gaussian for smoothing. Figure 16e,f shows the maps obtained using the proposed framework. The foveal lattice has five rings (rexel sizes ranging from 32 × 32 to 2 × 2) and a fovea of 176 × 140 pixels. The original images were slightly resized to 672 × 512 pixels, to make height and weight values divisible by 32. The segmentation parameters were heuristically set to obtain the better results on all of the Toronto database, but they remain the same for evaluating all of the images. The relevance of all regions provided by the segmenter was evaluated using a set of weights where each $\lambda_{i}=0.2$, and the obtained saliency map was smoothed by a Gaussian filter. This procedure is illustrated in Figure 17.

The quantitative evaluation of the proposal employs the area under the ROC (receiver operating characteristic) curve [31] for scoring. The area under the curve (AUC) considers eye fixations as the positive set and random points from the image as the negative set. To eliminate center-bias effects, these negative sets can be built by the union of all fixations of all subjects across all other images in the database, except for the positive set. Then, the saliency map is considered as a binary classifier to discriminate positive and negative samples. The ROC curve associated with each image on the database is obtained by thresholding over the saliency map and plotting true positive rate versus false positive rate. A global ROC curve averages the curves of all images, and the area underneath this final curve provides the final score. A score equal to one indicates a perfect prediction. Is it possible to reach this value? On the Toronto database, the density maps obtained from the fixations of one person provide a mean score of 0.878. This could be considered the real upper limit [31]. Using this evaluation framework, our proposal provides a final score of 0.669 on the Toronto database. Within the whole set of 28 models evaluated by Borji et al. [31], the method takes fifth place. The AWS [32] model is significantly better than all other models over this database, followed by the HouNIPS [33], AIM [34] and Judd [35] models. Only these approaches are over 0.675 (see [31] for further details).

**Figure 18:**
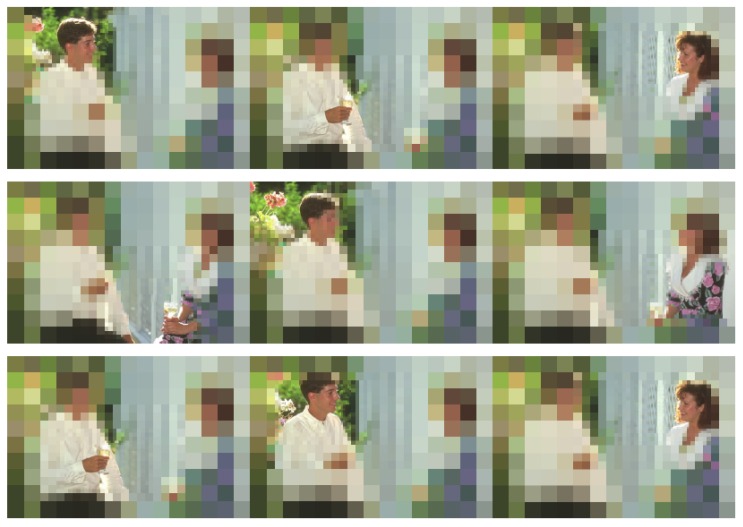
Active exploration of Image #157055 of the BSDS500: from the top-left corner to the bottom-right, the figure shows the sequence of fixations.

However, the goal of our proposal is to be able to actively explore a scene, moving the fovea from one location to a new one, while the whole scene is segmented and evaluated. This behavior is shown in Figure 18. For evaluating the ability of our proposal for exploring a scene using a foveal strategy, we have also tested the whole framework using the JUDDdatabase [35]. This dataset provides eye tracking data of 15 viewers on 1003 images. To compare with other approaches using this scheme, we will characterize each proto-object by its centroid. Thus, the sequence of proto-objects is converted into a sequence of positions. Using the similarity index proposed by Liu et al. [36], we can compare this sequence with a reference pattern. This metric employs a gap parameter, which penalizes when it is necessary to insert or remove a fixation on the trajectory during the matching of both sequences. Following the guidelines on this work, this gap has been set to −0.5 in our experiments. Finally, our scan path was compared to the 15 reference patterns (one per viewer). The mean value was 0.95, improving the results from other approaches, such as the one from Walther and Koch [37]. The score is under the 1.15 obtained by Liu et al. [36]. It must be noted that this approach combines low-level features with the properties of a higher significance level, which are not considered within our approach.

## 7. Discussion

The basis of active vision lays on the existence of a mechanism able to autonomously detect what is the most relevant region of interest in the scene. Thus, it is possible to direct the focus of attention on it. The goal is to maintain the impression that the entire scene is perceived in real time because a continuous stream of relevant parts of the image are explored and analyzed. This ‘what’ stream, which is not the topic covered in this paper, is complemented with a ‘where’ stream. Additionally, the feasibility of processing the whole scene is achieved by using a space-variant sampling of the whole field of view and a multiscale segmentation of this foveal lattice. This paper describes how both solutions are implemented on the different parts (programmable logic and processing system) of the Zynq AP SoC. The hardware-software division in our work responds to the nature of the modules. The ones in charge of providing the stream of foveal images are grouped into a subsystem and implemented in programmable logic. With the visual sensor, they successfully synthesize a camera that is able to provide a stream of foveal images without significant latency. These images are available for the ARM core in real time.

The segmentation of the sequence of foveal images has been accelerated by using a hierarchical approach. Our proposal is based on the D3P algorithm, but uses a foveal lattice to build the base level. This minor detail introduces two significant differences with respect to previous approaches: (i) the full FoV is encoded in all levels of the hierarchy; and (ii) the region of all levels associated with the fovea is better defined than the rest of the level. However, when using the original D3P, each vertex of the structure is linked to a very large number of neighbors. The memory resources and computation cost were excessive for running the algorithm in the ARM. With the aim of reducing both parameters, we designed the bounded D3P. The performances of the D3P and BD3P algorithms are practically the same, but the BD3P works significantly faster. This issue is illustrated in Table 2. The average height of the BD3P is almost half the one of the D3P. The segmenter divides up the image into regions, whose saliency is then estimated. The computation of the features employed for computing the saliency is also accelerated by using the rexel decomposition present at the base level of the hierarchy. Thus, the proposed object-based saliency model runs at 10 fps with images that cover a FoV of five megapixels at uniform resolution. The verification of the segmentation scheme was conducted using the BSDS500 database. It is significant to note that our approach never works with the whole uniform image. However, five foveations were sufficient to draw the significant contours on the image. According to the obtained precision-recall curve, there exist better approaches, but our approach only manages a compressed (i.e., foveated) version of the image. This allows our proposal to be very fast and to employ lesser memory resources. Finally, the validation of the whole framework was provided by testing it using the Toronto and JUDD databases. The parameters of the segmenter were maintained constant in all of these tests, and the obtained scores were similar to the ones provided by the best approaches. We can conclude that the approach is robust and able to deal with natural scenes (as these scenes are present in all tested databases (BSDS500, Toronto and JUDD)).

This paper mainly focuses on two lines of work, complementary to the design of the mechanism of gaze control: the design of the whole architecture and the concrete development of a hardware capable of generating foveal color images. We are currently working on including the segmenter in the programmable logic of the AP SoC. We also focus on improving the mechanism of attention, which currently does not include basic elements, such as an efficient inhibition of return [34]. This will imply migrating the solution to larger platforms, such as the Zynq UltraScale+ MPSoC series from Xilinx.

## Figures and Tables

**Table 1:** Estimated system resources and performance as reported by the synthesis tool (the max image size is 2592 × 1944 pixels, and the target clock period is 10 ns).

	Image Demosaicing	Foveal Mapping	rgb2hsv
**Digital Signal Processor (DSP)**	3	0	3
**Block Random Access Memory (BRAM_18K)**	6	18	0
**Flip-flop**	500	1227	3305
**LUT**	543	1242	3006
**Clock Period (ns)**	9.40	5.42	8.91
**Loop Latency (clocks)**	5,043,391	5,038,862	5,038,879
**Initiation Interval (clocks)**	1	1	1

**Table 2:** *Q* value and the number of levels of the pyramid for several decimation algorithms (see the text). LRP, linked regular pyramid; BIP, bounded irregular pyramid; HIP, hierarchy of partitions; CIP, the combinatorial pyramid.

	$Q_{\min}$	$Q_{\mathrm{ave}}$	$Q_{\max}$	$h_{\min}$	$h_{\mathrm{ave}}$	$h_{\max}$
**LRP**	1052.1	1570.3	2210.3	9	9	**9**
**WRP**	1133.7	1503.5	2080.8	9	9	**9**
**D3P**	355.6	**818.5**	1301.1	11	32.9	64
**BIP**	**343.2**	1090.9	1911.3	**8**	**8.7**	15
**HIP**	460.5	955.1	1530.7	9	11.4	19
**CIP**	430.7	870.2	**1283.7**	9	74.2	202
**BD3P**	412.6	831.5	1497.1	9	13	18
